# A β-cyclodextrin Modified Graphitic Carbon Nitride with Au Co-Catalyst for Efficient Photocatalytic Hydrogen Peroxide Production

**DOI:** 10.3390/nano10101969

**Published:** 2020-10-04

**Authors:** Guifu Zuo, Yuqian Zhang, Shanshan Liu, Zhaoliang Guo, Qiannan Zhao, Gopalan Saianand, Liwei Feng, Lijuan Li, Wangze Li, Ning Zhang, Xianguang Meng, Vellaisamy A. L. Roy

**Affiliations:** 1 Hebei Provincial Laboratory of Inorganic Nonmetallic Materials, College of Materials Science and Engineering, North China University of Science and Technology, Tangshan 063210, China; zuoguifu@163.com (G.Z.); 18332737639@163.com (Y.Z.); guozhaoliangcl@163.com (Z.G.); 18332725160@163.com (Q.Z.); 13473519550@163.com (L.F.); Lilj5527@163.com (L.L.); Liwangze2008@126.com (W.L.); 2 College of Materials Science and Engineering, Zhengzhou University, Zhengzhou 450001, China; 18712839227@163.com; 3 Global Centre for Environmental Remediation (GCER), Faculty of Science, The University of Newcastle, Callaghan, NSW 2308, Australia; saianand.gopalan@gmail.com; 4 School of Materials Science and Engineering, Central South University, Changsha 410083, China; nzhang@csu.edu.cn; 5 Department of Materials Science and Engineering, City University of Hong Kong, Tat Chee Avenue, Kowloon, Hong Kong 999077, China; 6 James Watt School of Engineering, University of Glasgow, Glasgow G12 8QQ, UK

**Keywords:** photocatalysis, oxygen reduction reaction, hydrogen peroxide, graphitic carbon nitride, β-cyclodextrin

## Abstract

Photocatalytic hydrogen peroxide (H_2_O_2_) production has attracted considerable attention as a renewable and environment-friendly method to replace other traditional production techniques. The performance of H_2_O_2_ production remains limited by the inertness of graphitic carbon nitride (CN) towards the adsorption and activation of O_2_. In this work, a photocatalyst comprising of β-cyclodextrin (β-CD)-modified CN with supporting Au co-catalyst (Au/β-CD-CN) has been utilized for effective H_2_O_2_ production under visible light irradiation. The static contact angle measurement suggested that β-CD modification increased the hydrophobicity of the CN photocatalyst as well as its affinity to oxygen gas, leading to an increase in H_2_O_2_ production. The rate of H_2_O_2_ production reached more than 0.1 mM/h under visible-light irradiation. The electron spin resonance spectra indicated that H_2_O_2_ was directly formed via a 2-electron oxygen reduction reaction (ORR) over the Au/β-CD-CN photocatalyst.

## 1. Introduction

Hydrogen peroxide (H_2_O_2_), is not only an oxidant but also an important product and/or a by-product in chemical and environmental protection [1,2,3,4,5]. At present, the anthraquinone method for industrial H_2_O_2_ production requires massive energy and organic solvents [6]. Compared with the anthraquinone method, the photocatalytic H_2_O_2_ production over semiconductors, such as TiO_2_ [7,8], ZnO [9,10], BiVO_4_ [11,12], and carbon nitride (CN) [13,14,15,16,17] is an ideal pathway that only uses solar light as driving force. In particular, metal-free CN has attracted great attention due to its visible light-responsive, environmentally friendly, and easy-synthesis features [18]. However, the photocatalytic activity of pure CN is still limited due to the recombination of charge carriers and poor activation ability of O_2_ for producing H_2_O_2_. An important strategy is loading proper co-catalyst on the surface of a photocatalyst to promote the charge separation and the activation of O_2_ [19,20]. Our previous study found that finely dispersed Au nanoparticles were excellent co-catalysts for improving the photocatalytic activity of CN toward enhancing the production of H_2_O_2_ [21]. The Au/CN system provided a promising prototype for producing concentrated H_2_O_2_ with an extremely low Au loading amount. In the liquid reaction system, the H_2_O_2_ production is limited by the adsorption of oxygen gas on photocatalyst because of the low solubility of oxygen gas in water and the inert surface nature of CN. Recently, we found a new method to further improve the performance of Au/CN for photocatalytic H_2_O_2_ synthesis by the modification of β-cyclodextrin (β-CD) on CN (β-CD-CN). The β-CD is a cyclic oligosaccharide with abundant oxygen-containing functional groups with a ring structure.Interestingly, the β-CD could significantly improve the hydrophobicity as well as oxygen adsorption of CN. Thus H_2_O_2_ production rate of Au/β-CD-CN under visible light irradiation was much higher than Au/CN. Simultaneously, the Au/β-CD-CN hybrid was very inert to catalyze the decomposition of H_2_O_2_, which was beneficial to the production of concentrated H_2_O_2_.

## 2. Materials and Methods

### 2.1. Preparation of Photocatalysts

#### 2.1.1. Preparation of g-C_3_N_4_

The g-C_3_N_4_ was prepared according to a reported procedure [22]. Dicyandiamide (Yongda Chemical Reagent Co., Ltd, Tianjin, China) was kept on a covered crucible and heated at a ramping rate of 3 °C/min up to 550 °C for 4 h in the air.

#### 2.1.2. Preparation of β-Cyclodextrin–Carbon Nitride (β-CD-CN) Composite

Typically, 12 g of β-CD (Yongda Chemical Reagent Co., Ltd, Tianjin, China) and 0.4228 g of NaOH (Yongda Chemical Reagent Co., Ltd, Tianjin, China) were mixed in 100 mL of water under vigorous stirring at 90 °C to remove the residual water. Then, the precipitate was ground into a fine powder. 8 g of treated β-CD was completely dissolved in N,N-Dimethylformamide (DMF) (Yongda Chemical Reagent Co., Ltd, Tianjin, China) at 90 °C. 1.5 mL of γ-(2,3-epoxypropoxy) propyl trimethoxysilane (KH-560) (Guangfu Fine Chemical Research Institute, Tianjin, China) was continuously added and stirred for 6 h. Then, 4 g of CN was put into the above solution and stirred for 12 h. The sample was thoroughly washed with distilled water and dried at 80 °C for 12 h.

#### 2.1.3. Preparation of Metal Co-Catalyst/β-CD-CN Nanocomposite

HAuCl_4_·4H_2_O (Guangfu Fine Chemical Research Institute, Tianjin, China) (335.8 μL, 0.0243 mol/L) was added to 250 mL of water containing β-CD-CN (2.5 g). The water was volatilized at 70 °C under vigorous stirring. The obtained sample reacted with KBH_4_ (Guangfu Fine Chemical Research Institute, Tianjin, China) to reduce HAuCl_4_·4H_2_O under vigorous stirring for 3 h. Then, the sample was centrifuged and washed with distilled water. The sample was designated as 0.05% Au/β-CD-CN. Accordingly, 0.03%, 0.08%, and 0.1% Au/β-CD-CN were obtained by the same procedure. For a fair comparison, 0.05% Pt/β-CD-CN, Ag/β-CD-CN, and Pd/β-CD-CN were prepared as above using H_2_PtCl_6_·6H_2_O, AgNO_3_, and PdCl_2_ (Guangfu Fine Chemical Research Institute, Tianjin, China), respectively.

### 2.2. Material Characterization

X-ray diffraction (XRD) data were obtained on an X-ray diffractometer (Rigaku SmartLab, Tokyo, Japan) operating with Cu Kα radiation. Energy-dispersive spectroscopy (EDS) was recorded by a scanning electron microscope (SEM) (FEI Scios, OR, USA) to analyze the elemental compositions of Au/β-CD-CN. TEM observations were performed using a scanning transmission electron microscope (STEM) (JEOL JEM-ARM200F, Tokyo, Japan) operated at 200 kV.Ultraviolet–visible (UV–vis) spectra were collected by Lambda 750S UV–vis spectrometer (Perkin-Elmer, Waltham, MA, USA) with an integrating sphere scanning from 300 to 600 nm. Photoluminescence spectra were obtained with a fluorescence spectrometer (PerkinElmer LS55, Waltham, MA, USA). The static contact angle (water) was tested with the contact angle system ((DH-HV1351UM, Shanghai Zhongchen Digital Technology Equipment Co., Ltd., Shanghai, China).

### 2.3. Photocatalytic Reactivity Test and H_2_O_2_ Decomposition

Typically, photocatalyst (0.4 g) was put into 100 mL of an aqueous solution including 10 mL of C_2_H_5_OH (sacrificial agent) to evaluate H_2_O_2_ production according to the literature [21]. The system was ultrasonicated for 3 min. The temperature of the reaction solution was cooled by the flow of cooling water which keep the system at room temperature. A 300 W Xe visible lamp with a UVCUT420 filter (λ > 420 nm) (PLS-SXE300, Bofeilai Technology Co., Ltd, Beijing, China) was used as visible light source with a constant flow of O_2_. The generated H_2_O_2_ was measured by a spectrophotometric method, using 2,9-dimethyl-1,10-phenanthroline (DMP) (Wengjiang Chemical Reagent Co., Ltd, Guangdong, China) and copper (II) ion (Guangfu Fine Chemical Research Institute, Tianjin, China) [23].

The decomposition test of H_2_O_2_ was similar to H_2_O_2_ production. The photocatalyst (0.4 g) and aqueous solution (ethanol:water = 1:9) were mixed in the reaction cell with H_2_O_2_ (Yongda Chemical Reagent Co., Ltd, Tianjin, China) (3500 μM). A 300 W Xe visible lamp was chosen as an irradiation source with a constant flow of N_2_ supply.

### 2.4. Electron Spin Resonance (ESR) Test

Electron spin resonance (ESR) analysis with 5,5-dimethyl-1-pyrroline N-oxide (DMPO) (Wengjiang Chemical Reagent Co., Ltd, Guangdong, China) as a spin-trapping reagent was performed to confirm the pathway of O_2_ reduction over different catalysts. ESR signals of radicals trapped by DMPO were detected with a ESP 300E spectrometer (Brucker, Faellanden, Switzerland). Typically, catalyst (20 mg) was added to an alcohol/water mixture (1/9 *v*/*v*, 5 mL) containing DMPO (0.125 mmol) within a container. After O_2_ bubbling for 3 min, the container was photo irradiated for 5 min. The catalyst was recovered by filtration, and the solution was subjected to analysis at room temperature.

## 3. Results and Discussion

### 3.1. Material Characterization

#### 3.1.1. Surface Morphology Characterization

**Figure 1:**
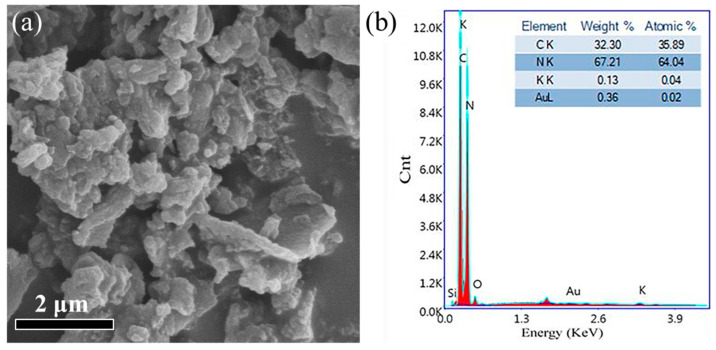
(**a**) Scanning electron microscopy (SEM) image of 0.05% Au/β-cyclodextrin–carbon nitride (β-CD-CN); (**b**) energy-dispersive spectroscopy (EDS) spectra of 0.05% Au/β-CD-CN.

The SEM image shows that the as-developed (0.05% Au/β-CD-CN) has an irregular layer structure (Figure 1a). The EDS result in Figure 1b demonstrates the co-existence of C, N, O, and Au atoms over the Au/β-CD-CN. The O atom shown in EDS is attributed to the β-CD. The actual Au content of 0.03%, 0.05%, 0.08%, and 0.1% Au/β-CD-CN samples were found to be 0.013%, 0.047%, 0.085%, and 0.106% through inductively coupled plasma mass spectrometry (ICP-MS) (Table S1).

**Figure 2:**
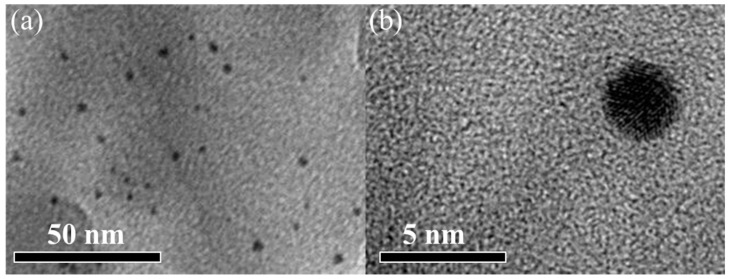
(**a**) Low and (**b**) high magnification STEM image of 0.05% Au/β-CD-CN.

The STEM images (high resolution and low resolution) of 0.05% Au/β-CD-CN are shown in Figure 2. It can be found that Au particles are finely dispersed on the surface of β-CD-CN. The average size of Au nanoparticles is about 3 nm in diameters.

#### 3.1.2. X-Ray Diffraction (XRD) and Fourier Transform Infrared (FT-IR) Characterizations

**Figure 3:**
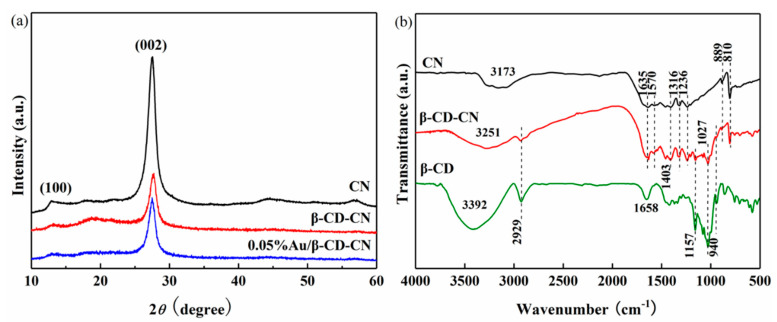
(**a**) X-ray diffraction (XRD) patterns of CN, β-CD-CN, and 0.05% Au/β-CD-CN; (**b**) Fourier transform infrared (FT-IR) spectra of CN, β-CD-CN, and β-CD.

All investigated samples show X-ray diffraction (XRD) patterns which can be obviously seen at 12.8° and 27.4°, indicating that they all have a similar crystal structure to CN (Figure 3a) [22]. The peak at 12.8° is related to the crystal plane of (100), which is attributed to the existence of an oxazine ring network. The peak at 27.4° corresponds to the characteristic interlayer stacking reflection of conjugated aromatic systems. As for Au, its content is relatively low, thus its diffraction peaks cannot be observed directly [24,25].

Figure 3b shows the Fourier transform infrared (FT-IR) spectra of the as-prepared CN, β-CD-CN, and β-CD. The original CN shows typical characteristic peaks as reported [26]. The peak at 810 cm^−1^ can be correlated to the vibrational absorptions of tri-s-triazine subunits. The typical absorption peaks of CN at 1570 cm^−1^ and 1635 cm^−1^ correspond to C–N and C=N, respectively. The vibration bands at 1236 cm^−1^, 1316 cm^−1^, and 1403 cm^−1^ belong to the aromatic C–N heterocycles. The formation of a broad peak at 3173 cm^−1^ is linked to N–H stretching. All the above peaks arise in the β-CD-CN. After CN modification by β-CD, new adsorption bands at around 550 cm^−1^, 1027 cm^−1^, and 1157 cm^−1^ are related to the β-phase formation, C–O–C, and C–C/C–O. Those are attributed to a large number of hydroxyl groups of β-CD [27,28,29]. Due to the asymmetric stretching vibration of C–H, an absorption peak appears at 2925 cm^−1^. The broad vibration band at 3392 cm^−1^ belongs to O–H stretching vibration, which also arises in the spectra of the pure β-CD sample. These results clearly indicate the formation of β-CD-CN [30].

#### 3.1.3. The Optical Absorption, Photoluminescence and Surface Properties

**Figure 4:**
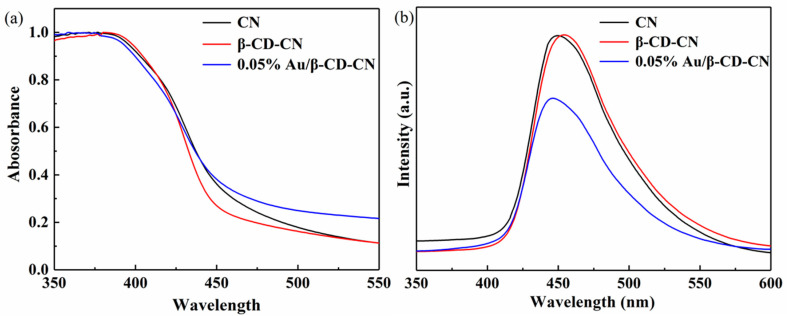
(**a**) Ultraviolet–visible (UV–vis) diffuse-reflectance spectra and (**b**) photoluminescence (PL) spectra of CN, β-CD-CN, and 0.05% Au/β-CD-CN.

The optical absorption of CN, β-CD-CN, and 0.05% Au/β-CD-CN were measured by UV–vis. All samples show wide absorption in the visible region (Figure 4a). The absorption edge of β-CD-CN is slightly shifted to shorter wavelengths due to the formation of a hydrogen bond between the amino group of CN and the hydroxyl group of β-CD. The hydrogen bond can improve charge separation kinetics and promote photocatalytic efficiency [31]. Compared with the β-CD-CN, a wide absorption in the range of 375–450 nm can be observed for Au/β-CD-CN, which is caused by the surface plasmon effect of Au in β-CD-CN [20,32,33].

The photoluminescence (PL) spectra of CN, β-CD-CN, and 0.05% Au/β-CD-CN nanocomposites are presented in Figure 4b. The emission peak at 455 nm is related to the band-band PL phenomenon of CN [34]. Compared with CN and β-CD-CN, the PL intensity of 0.05% Au/β-CD-CN is significantly reduced. It is well documented [35] that the PL intensity can be used to characterize the recombination of photogenerated electron-hole pairs. The obvious fluorescence quenching of 0.05% Au/β-CD-CN is owing to the efficient separation of charge carriers which is beneficial for increasing H_2_O_2_ production [36].

**Figure 5:**
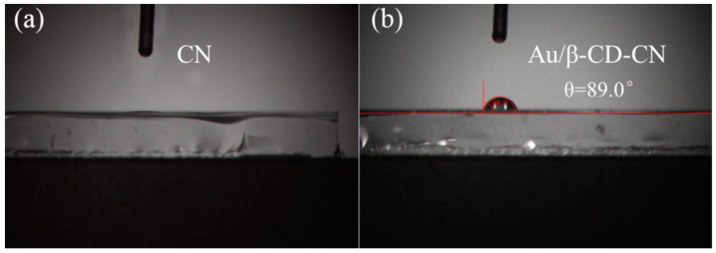
The water contact angle of CN (**a**) and Au/β-CD-CN (**b**).

A water contact angle test was carried out to study the change in the Au/β-CD-CN surface property. As shown in Figure 5, it is obvious that the β-CD modified CN has a hydrophobic surface with a contact angle of about 89.0°, leading to better adsorption ability to oxygen and thus improved photocatalytic activity of H_2_O_2_ production [37].

### 3.2. Photocatalytic Performance

#### 3.2.1. Photocatalytic H_2_O_2_ Production Activity

**Figure 6:**
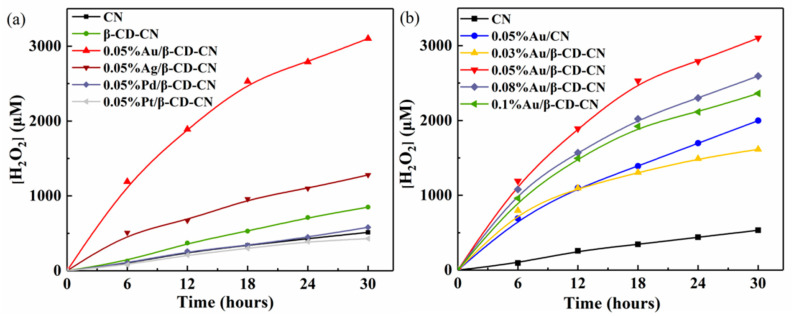
(**a**) Photocatalytic H_2_O_2_ production over different co-catalysts loaded CN and (**b**) Au/β-CD-CN with different loading amounts of Au (λ > 420 nm).

The photocatalytic activity of H_2_O_2_ production over M/β-CD-CN composite was tested under visible light and a spectrophotometric method was used to quantify the H_2_O_2_ concentrations [37]. The standard curve indicated that the concentration of H_2_O_2_ and absorbance exhibited a good linear relationship with R-square of 0.9988 (Figure S1). The photocatalytic activity of β-CD-CN with different co-catalysts for H_2_O_2_ production was as follows (Figure 6). The β-CD modified CN exhibited excellent photocatalytic activity than CN for H_2_O_2_ production. Furthermore, the Au and Ag enhanced photocatalytic activity remarkably for H_2_O_2_ production. β-CD-CN with Au loading amount (0.05%) showed the best photocatalytic activity for H_2_O_2_ production and the reaction rate reached more than 0.1 mM/h. Moreover, the amount of Au loading on β-CD-CN is critical to the photocatalytic activity (Figure 6b). When the amount of Au loading exceeds 0.05% on β-CD-CN, the photocatalytic activity decreased. The high load of Au possibly hindered the light absorption capability of CN and hence results in decreased photocatalytic activity [38].

#### 3.2.2. Photocatalytic H_2_O_2_ Decomposition Activity

**Figure 7:**
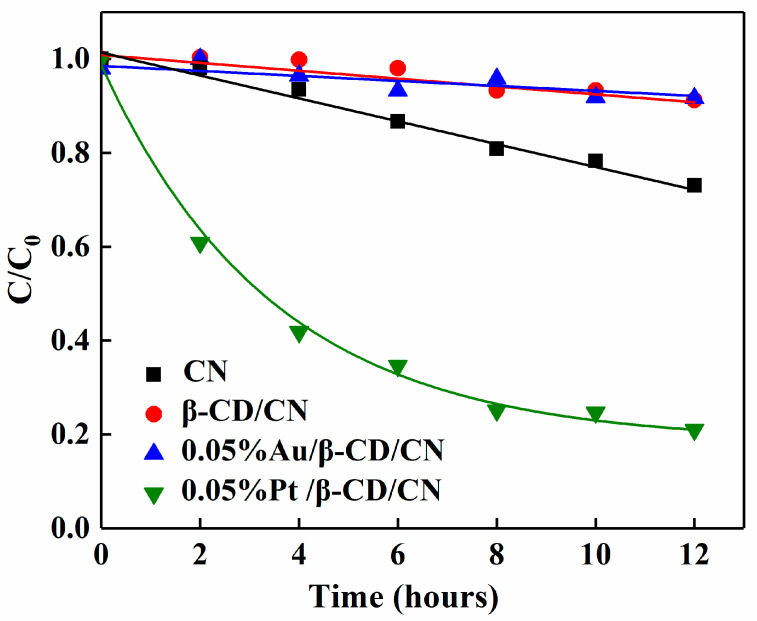
Photodecomposition of H_2_O_2_ in an aqueous CN suspension with a different metal co-catalyst.

The H_2_O_2_ formation is generally accompanied by H_2_O_2_ decomposition which results in lower H_2_O_2_ production [6,13]. Therefore, it is crucial to restrain the decomposition process to keep H_2_O_2_ evolution as sustainable as possible. The results of H_2_O_2_ photodecomposition in aqueous suspension with different photocatalysts are shown in Figure 7. The Au/β-CD-CN is inert for the decomposition of H_2_O_2_, which is also the reason for its high H_2_O_2_ production activity. However, the Pt/β-CD-CN promotes H_2_O_2_ decomposition via dissociative adsorption and results in a high decomposition rate [39]. Apparently, the H_2_O_2_ decomposition curves of the Au-loaded sample displayed a slow zero-order kinetic process, while the Pt-loaded sample displayed a fast first-order kinetic process.

### 3.3. Plausible Mechanism of Photocatalysis

**Figure 8:**
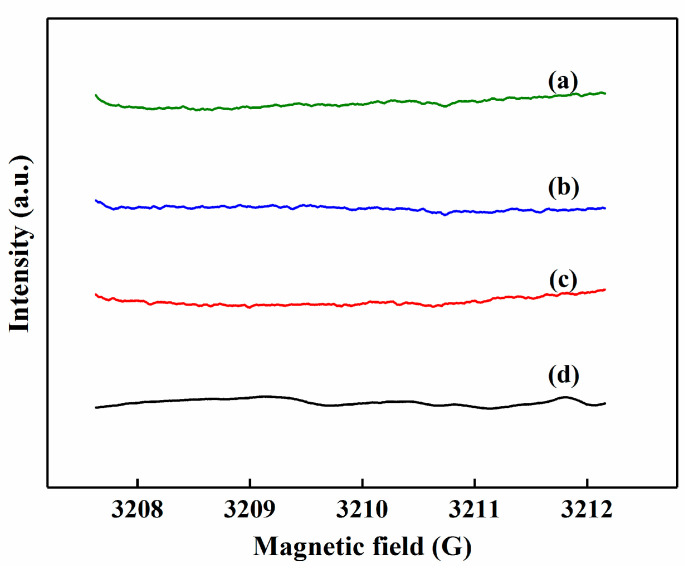
In situ electron spin resonance (ESR) spectra of photocatalytic ORR over different photocatalysts under visible light irradiation. Photocatalysts: (**a**) CN, (**b**) β-CD-CN, (**c**) 0.05%Au/β-CD-CN, (**d**) 0.05%Pt/β-CD-CN.

To study the reaction mechanism of H_2_O_2_ production over the photocatalysts, the ESR analysis results are shown in Figure 8. It can be seen that neither the ·OOH signal nor the ·OH signal was observed in the process of H_2_O_2_ production, indicating that H_2_O_2_ was formed via direct 2-electron oxygen reduction over photocatalysis [40].

**Figure 9:**
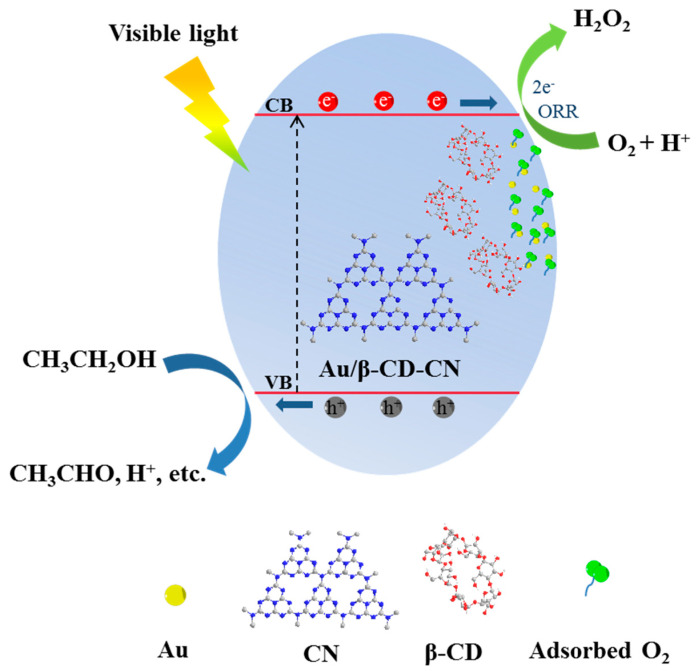
Proposed mechanism of photocatalytic H_2_O_2_ production over Au/β-CD-CN composite under visible light.

The mechanism of Au/β-CD-CN photocatalytic activity of H_2_O_2_ is shown in Figure 9. When irradiated by visible light, electrons (e^−^) and holes (h^+^) are separated (Equation (1)), e^−^ is positioned at the C1 and N4 positions of the triazine ring, and h^+^ is positioned at the N2 and N6 positions, that is, an electron (e^−^)-hole (h^+^) pair is formed. However, electrons easily recombine with holes. After CN is loaded with Au, electrons (e^−^) can be rapidly transferred from C1 and N4 to Au, thereby effectively inhibiting the recombination of electrons and holes. Holes (h^+^) capture α-H and β-H in CH_3_CH_2_OH, and CH_3_CH_2_OH generates CH_3_CHO (Equation (2)). Immediately after β-CD modification, a hydrogen bond formed between the amino group of CN and the hydroxyl group of β-CD. The β-CD-CN plays an excellent adsorption role with oxygen. Electrons (e^−^) reduce O_2_ to produce H_2_O_2_ and complete the photocatalytic cycle (Equation (3)).

g-C_3_N_4_ → e^−^ + h^+^ (1)

CH_3_CH_2_OH + 2h^+^ → CH_3_CHO + 2H^+^ (2)

O_2_ + 2H^+^ + 2e^−^ → H_2_O_2_ (3)

## 4. Conclusions

Au/β-CD-CN was successfully obtained for improving photocatalytic activity for H_2_O_2_ production under visible light. The β-CD modified CN showed a hydrophobic property, which was beneficial for adsorption with oxygen to improve H_2_O_2_ production. Significantly, the optimal photocatalyst with the Au loading amount as low as 0.05% reached the maximal H_2_O_2_ production rate. Simultaneously, Au/β-CD-CN exhibited an inertness decomposition rate of H_2_O_2_ under visible light. The ESR test demonstrated that H_2_O_2_ was directly formed via 2-electron oxygen reduction over photocatalysis. This article provides a new method that loading Au on β-CD-modified CN can increase the adsorption and activation of O_2_ to improve photocatalytic activity for H_2_O_2_ production.

## Supplementary Materials

The following are available online at https://www.mdpi.com/2079-4991/10/10/1969/s1: Table S1: Inductively coupled plasma mass spectrometry (ICP-MS) test results of Au/β-CD-CN with different Au content, Figure S1: Standard curve: a linear relationship for the optical absorbance at 454 nm as a function of H_2_O_2_ concentration.

## References

[1] Yusuke Y., Akifumi N., Takamitsu M., Kei O., Shunichi F. (2013). Acetate induced enhancement of photocatalytic hydrogen peroxide production from oxalic acid and dioxygen. J. Phys. Chem. A.

[2] Lee K.P., Gopalan A.I., Komathi S. (2009). Direct electrochemistry of cytochrome c and biosensing for hydrogen peroxide on polyaniline grafted multi-walled carbon nanotube electrode. Sens. Actuators B Chem..

[3] Gopalan A.I., Komathi S., Anand G.S., Lee K.P. (2013). Nanodiamond based sponges with entrapped enzyme: A novel electrochemical probe for hydrogen peroxide. Biosens. Bioelectron..

[4] Komathi S., Gopalan A.I., Kim S.K., Anand G.S., Lee K.P. (2013). Fabrication of horseradish peroxidase immobilized poly(N-[3-(trimethoxy silyl)propyl]aniline) gold nanorods film modified electrode and electrochemical hydrogen peroxide sensing. Electrochim. Acta.

[5] Bui Q.T., Yu I.K., Gopalan A.L., Saianand G., Kim W., Choi S.H. (2019). Facile Fabrication of Metal Oxide Based Catalytic Electrodes by AC Plasma Deposition and Electrochemical Detection of Hydrogen Peroxide. Catalysts.

[6] Moon G.H., Kim W., Bokare A.D., Sung N.E., Choi W. (2014). Solar production of H_2_O_2_ on reduced graphene oxide-TiO_2_ hybrid photocatalysts consisting of earth-abundant elements only. Energy Environ. Sci..

[7] Zuo G.F., Li B.D., Guo Z.L., Wang L., Yang F., Hou W.S., Zhang S.T., Zong P.X., Liu S.S., Meng X.G. (2019). Efficient Photocatalytic Hydrogen Peroxide Production over TiO_2_ Passivated by SnO_2_. Catalysts.

[8] Miwako T., Shin-Ichi N., Hiroaki T. (2010). In situ liquid phase synthesis of hydrogen peroxide from molecular oxygen using gold nanoparticle-loaded titanium(IV) dioxide photocatalyst. J. Am. Chem. Soc..

[9] Meng X.G., Zong P.X., Wang L., Yang F., Hou W.S., Zhang S.T., Li B.D., Guo Z.L., Liu S.S., Zuo G.F. (2020). Au-nanoparticle-supported ZnO as highly efficient photocatalyst for H_2_O_2_ production. Catal. Commun..

[10] Domènech X., Ayllón J.A., Peral J. (2001). H_2_O_2_ Formation from photocatalytic processes at the ZnO/water interface. Environ. Sci. Pollut. R..

[11] Chen S.H., Jiang Y.S., Lin H.Y. (2020). Easy Synthesis of BiVO_4_ for Photocatalytic Overall Water Splitting. ACS Omega.

[12] Hirakawa H., Shiota S., Shiraishi Y., Sakamoto H., Hirai T. (2016). Au Nanoparticles Supported on BiVO_4_: Effective Inorganic Photocatalysts for H_2_O_2_ Production from Water and O_2_ under Visible Light. ACS Catal..

[13] Yang L., Dong G., Jacobs D.L., Wang Y., Zang L., Wang C. (2017). Two-channel photocatalytic production of H_2_O_2_ over g-C_3_N_4_ nanosheets modified with perylene imides. J. Catal..

[14] Peng Y.L., Wang L.Z., Liu Y.D., Chen H.J., Lei J.Y., Zhang J.L. (2017). Visible-light-driven photocatalytic H_2_O_2_ production on g-C_3_N_4_ loaded with CoP as a noble metal free cocatalyst. Eur. J. Inorg. Chem..

[15] Xiong W., Huang F., Zhang R.Q. (2020). Recent developments on carbon nitride based films for photoelectrochemical water splitting. Sustain. Energy Fuels.

[16] Wu C.B., Yu G.H., Yin Y., Wang Y.Z., Chen L., Han Q., Tang J.W., Wang B. (2020). Mesoporous Polymeric Cyanamide-Triazole-Heptazine Photocatalysts for Highly-Efficient Water Splitting. Small.

[17] Chi H.L., Liu J.X., Zhang X.Y., Xue X.G., Zhang D.D., Lin X.C., Huang P.R., Sun L.X., Xiong J., Cai P. (2020). Synergetic defects boost charge separation in CN for enhanced photocatalytic water splitting. J. Mater. Chem. C.

[18] Maeda K., Wang X., Nishihara Y. (2009). Photocatalytic Activities of Graphitic Carbon Nitride Powder for Water Reduction and Oxidation under Visible Light. J. Phys. Chem. C.

[19] Meng X.G., Liu L.Q., Ouyang S.X., Xu H., Wang D.F., Zhao N.Q., Ye J.H. (2016). Nanometals for Solar-to-Chemical Energy Conversion: From Semiconductor-Based Photocatalysis to Plasmon-Mediated Photocatalysis and Photo-Thermocatalysis. Adv. Mater..

[20] Samanta S., Martha S., Parida K. (2014). Facile Synthesis of Au/g-C_3_N_4_ Nanocomposites: An Inorganic/Organic Hybrid Plasmonic Photocatalyst with Enhanced Hydrogen Gas Evolution Under Visible-Light Irradiation. ChemCatChem.

[21] Zuo G.F., Liu S.S., Wang L., Song H., Zong P.X., Hou W.S., Li B.D., Guo Z.L., Meng X.G., Du Y. (2019). Finely dispersed Au nanoparticles on graphitic carbon nitride as highly active photocatalyst for hydrogen peroxide production. Catal. Commun..

[22] Liu G.G., Zhao G.X., Wei Z., Liu Y.Y., Hong P., Zhang H.B., Dong H., Meng X.G., Peng L., Kako T. (2016). In Situ Bond Modulation of Graphitic Carbon Nitride to Construct p–n Homojunctions for Enhanced Photocatalytic Hydrogen Production. Adv. Funct. Mater..

[23] Kosaka K., Yamada H., Matsui S., Echigo S., Shishida K. (1998). Comparison among the Methods for Hydrogen Peroxide Measurements To Evaluate Advanced Oxidation Processes:  Application of a Spectrophotometric Method Using Copper(II) Ion and 2,9-Dimethyl-1,10-phenanthroline. Environ. Sci. Technol..

[24] Jirkovsky J.S., Itai P., Elisabet A., Matej H., Simon R., Schiffrin D.J. (2011). Single atom hot-spots at Au-Pd nanoalloys for electrocatalytic H_2_O_2_ production. J. Am. Chem. Soc..

[25] Samira S., Arnau V.C., Mohammadreza K., Davide D., Paolo M., Björn W., María E.E., Paoli E.A., Rasmus F., Hansen T.W. (2013). Enabling direct H_2_O_2_ production through rational electrocatalyst design. Nat. Mater..

[26] Liu S.S., Dong W., Zeng X.F., Guo Z.L., Zong P.X., Li B.D., Meng X.G., Zuo G.F. (2019). β-cyclodextrin modified g-C_3_N_4_ nanosheet: A fluorescent drug carrier with ultrahigh drug loading capacity and pH-responsive release. J. Chem. Technol. Biot..

[27] Salimi A., Yousefi A.A. (2003). Analysis Method: FTIR studies of β -phase crystal formation in stretched PVDF films. Polym. Test..

[28] Song W.C., Hu J., Zhao Y., Shao D.D., Li J.X. (2013). Efficient removal of cobalt from aqueous solution using β-cyclodextrin modified graphene oxide. RSC Adv..

[29] Banerjee S.S., Chen D.H. (2008). Cyclodextrin conjugated magnetic colloidal nanoparticles as a nanocarrier for targeted anticancer drug delivery. Nanotechnology.

[30] Xu X.Y., Shang H., Zhang T.Y., Shu P.J., Liu Y.P., Xie J., Zhang D.Y., Tan H., Li J.S. (2018). A stimuli-responsive insulin delivery system based on reversible phenylboronate modified cyclodextrin with glucose triggered host-guest interaction. Int. J. Pharm..

[31] Ge L. (2011). Synthesis and photocatalytic performance of novel metal-free g-C_3_N_4_ photocatalysts. Mater. Lett..

[32] Gopalan S.A., Gopalan A.I., Vinu A., Lee K.P., Kang S.W. (2018). A new optical-electrical integrated buffer layer design based on gold nanoparticles tethered thiol containing sulfonated polyaniline towards enhancement of solar cell performance. Sol. Energy Mat. Sol. Cells.

[33] Anand G.S., Gopalan A.I., Kang S.W., Lee K.P. (2013). Development of a surface plasmon assisted label-free calorimetric method for sensitive detection of mercury based on functionalized gold nanorods. J. Anal. Atom. Spectrom..

[34] Lin L., Ou H.H., Zhang Y., Wang X. (2016). Tri-s-triazine-based Crystalline Graphitic Carbon Nitrides for Highly Efficient Hydrogen Evolution Photocatalysis. ACS Catal..

[35] Tahir M., Amin N.S. (2013). Photocatalytic reduction of carbon dioxide with water vapors over montmorillonite modified TiO_2_ nanocomposites. Appl. Catal. B-Environ..

[36] Liu Z., Hou W., Pavaskar P., Aykol M., Cronin S.B. (2011). Plasmon resonant enhancement of photocatalytic water splitting under visible illumination. Nano Lett..

[37] Feng D.X., Nguyen A.V., Tong X. (2017). Effect of contact angle and contact angle hysteresis on the floatability of spheres at the air-water interface. Adv. Colloid Interface Sci..

[38] Kuriki R., Matsunaga H., Nakashima T., Wada K., Yamakata A., Ishitani O., Maeda K. (2016). Nature-Inspired, Highly Durable CO_2_ Reduction System Consisting of a Binuclear Ruthenium(II) Complex and an Organic Semiconductor Using Visible Light. J. Am. Chem. Soc..

[39] Balbuena P.B., Calvo S.R., Lamas E.J., Salazar P.F., Seminario J.M. (2006). Adsorption and Dissociation of H_2_O_2_ on Pt and Pt-Alloy Clusters and Surfaces. J. Phys. Chem. B.

[40] Shiraishi Y., Kanazawa S., Sugano Y., Tsukamoto D., Sakamoto H., Ichikawa S., Hirai T. (2014). Highly Selective Production of Hydrogen Peroxide on Graphitic Carbon Nitride (g-C_3_N_4_) Photocatalyst Activated by Visible Light. ACS Catal..

