# Supplementary material for: A β-cyclodextrin Modified Graphitic Carbon Nitride with Au Co-Catalyst for Efficient Photocatalytic Hydrogen Peroxide Production
Source: Nanomaterials (Basel). 2020 Oct 4;10(10):1969. doi: 10.3390/nano10101969 (PMC7600220; doi:10.3390/nano10101969)
Supplement: Supplementary file 1 [file nanomaterials-10-01969-s001.pdf]

nanomaterials

Supplementary Materials

A β-cyclodextrin Modified Graphitic Carbon Nitride with Au Co-Catalyst for Efficient Photocatalytic Hydrogen Peroxide Production

Guifu Zuo [1], Yuqian Zhang [1], Shanshan Liu [2], Zhaoliang Guo [1], Qiannan Zhao [1], Gopalan Saianand [3], Liwei Feng [1], Lijuan Li [1], Wangze Li [1], Ning Zhang [4], Xianguang Meng [1,5,*] and Vellaisamy A.L. Roy [6,*]

[1] Hebei Provincial Laboratory of Inorganic Nonmetallic Materials, College of Materials Science and Engineering, North China University of Science and Technology, Tangshan 063210, China; zuoguifu@163.com (G.Z.); 18332737639@163.com (Y.Z.); guozhaoliangcl@163.com (Z.G.); 18332725160@163.com (Q.Z.); 13473519550@163.com (L.F.); Lilj5527@163.com (L.L.); Liwangze2008@126.com (W.L.)

[2] College of Materials Science and Engineering, Zhengzhou University, Zhengzhou 450001, China; 18712839227@163.com

[3] Global Centre for Environmental Remediation (GCER), Faculty of Science, The University of Newcastle, Callaghan 2308, New South Wales, Australia; saianand.gopalan@gmail.com

[4] School of Materials Science and Engineering, Central South University, Changsha, Hunan 410083, China; nzhang@csu.edu.cn

[5] Department of Materials Science and Engineering, City University of Hong Kong, Tat Chee Avenue, Kowloon, Hong Kong 999077, China

[6] James Watt School of Engineering, University of Glasgow, G12 8QQ, UK

* Correspondence: mengxg_materchem@163.com (X.M.); Roy. Vellaisamy@glasgow.ac.uk (V.A.L.R.)

Table S1. ICP-MS test results of Au/β-CD-CN with different Au content.

Samples	Theoretical content (%)	Actual content (%)
1	0.03	0.013
2	0.05	0.047
3	0.08	0.085
4	0.10	0.106

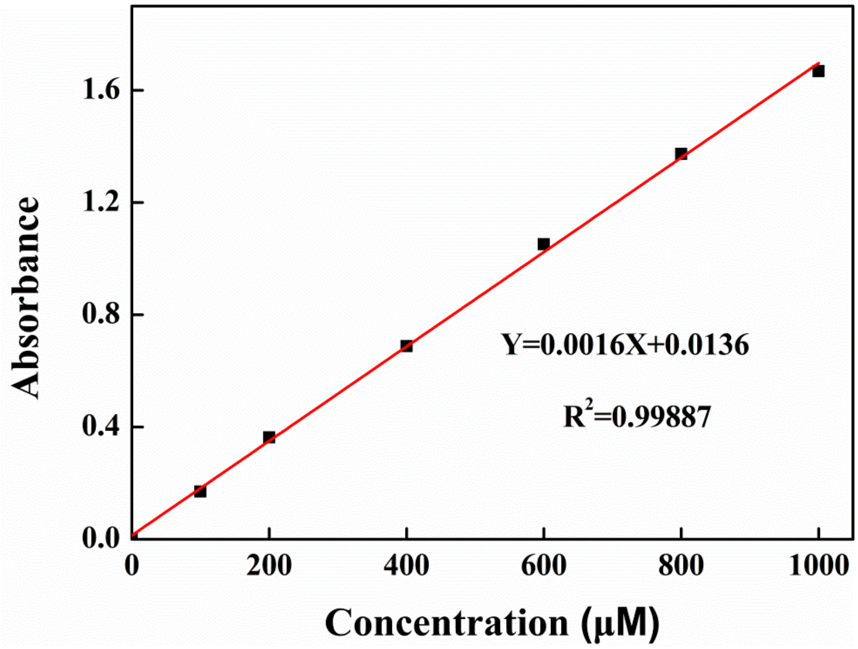

Figure S1. Standard curve: a linear relationship for the optical absorbance at 454 nm as a function of H_2O_2 concentration.
